# Intratumoral microbiota and their emerging role in the tumor microenvironment

**DOI:** 10.20517/mrr.2026.18

**Published:** 2026-09-22

**Authors:** Xianfeng Hui, Shihuan Ding, Ge Gao, Shuoxiang Gao, Jiaqi Tian, Shuochen Xu, Bingqi Yan, Rujing Zhang, Tiesuo Zhao, Hui Wang

**Affiliations:** ^1^Department of Immunology, School of Basic Medical Sciences, Henan Medical University, Xinxiang 453003, Henan, China.; ^2^Henan Key Laboratory of Immunology and Targeted Drug, Henan Medical University, Xinxiang 453003, Henan, China.; ^3^Xinxiang Engineering Technology Research Center of Immune Checkpoint Drug for Liver-Intestinal Tumors, Henan Medical University, Xinxiang 453003, Henan, China.; ^4^College of Medicine, Henan University, Kaifeng 475001, Henan, China.; ^5^Henan Collaborative Innovation Center of Molecular Diagnosis and Laboratory Medicine, School of Medical Technology, Henan Medical University, Xinxiang 453003, Henan, China.; ^#^These authors contributed equally to this work.

**Keywords:** Microorganisms within tumors, tumor microenvironment, immune regulation, treatment response, precision treatment

## Abstract

Intratumoral microbiota, as an important component of the tumor microenvironment, have attracted increasing attention in recent years for their roles in tumor initiation, progression, and therapeutic regulation. Accumulating evidence indicates that low-abundance yet relatively stable microbial communities are present in a variety of solid tumors, exhibiting marked tumor-type specificity and spatial heterogeneity. Current studies suggest that intratumoral microorganisms contribute to tumor progression through multiple mechanisms, including activation of oncogenic signaling pathways, modulation of chronic inflammatory responses, remodeling of the immunosuppressive microenvironment, and regulation of metabolic reprogramming and drug responsiveness. Meanwhile, their bidirectional effects on chemotherapy, radiotherapy, targeted therapy, and immunotherapy highlight their potential roles in determining therapeutic sensitivity and the development of resistance. Despite existing challenges such as low microbial biomass, contamination control, and difficulties in functional validation, advances in spatial multi-omics and single-cell technologies are continuously enhancing the potential clinical value of intratumoral microbiota in tumor diagnosis, prognostic assessment, and precision therapy. This review summarizes the sources, colonization mechanisms, biological functions, roles in therapeutic modulation, and clinical applications of intratumoral microbiota, aiming to provide a theoretical basis for future research and clinical translation.

## INTRODUCTION

Intratumoral microbiota has recently emerged as a key concept in tumor biology, referring to low-abundance microbial communities residing within the tumor microenvironment and their ecological features. Solid tumors were long considered “sterile,” largely due to the limited sensitivity of conventional culture methods and the predominant focus on tumor cell-intrinsic alterations. Within this framework, microorganisms were primarily implicated in infection-associated cancers - such as Helicobacter pylori in gastric cancer, human papillomavirus in cervical cancer^[1-3]^, and hepatitis B and C viruses in hepatocellular carcinoma^[4]^ - and were viewed as exogenous carcinogens rather than integral components of the tumor microenvironment.

Advances in high-throughput sequencing and molecular profiling have revealed important roles for microbiota in immune regulation and tumorigenesis. In particular, studies of the gut microbiota have demonstrated its involvement in colorectal cancer development and responses to immunotherapy. For example, enterotoxigenic Bacteroides fragilis (ETBF), a gut-associated bacterium, promotes colorectal tumorigenesis through B. fragilis toxin-induced Th17-mediated inflammation^[5]^. However, these early studies primarily focused on distal microbial ecosystems, and direct evidence demonstrating the presence and functional roles of endogenous microorganisms within tumor tissues remained limited.

Subsequent findings - including the detection of Fusobacterium nucleatum in colorectal cancer and bacterial DNA in pancreatic and other tumors - suggested that microbes may directly inhabit tumor tissues^[6-9]^. Yet, the extremely low microbial biomass and susceptibility to contamination rendered these findings controversial. This uncertainty was largely resolved by studies published between 2019 and 2020, which, using large cohorts, stringent controls, and *in situ* approaches, demonstrated the widespread presence of intratumoral microbiota across multiple cancer types, with tumor-specific signatures and distinct spatial organization. Notably, in pancreatic ductal adenocarcinoma, higher microbial diversity correlates with prolonged survival and enhanced antitumor immunity, as validated by fecal microbiota transplantation in preclinical models^[10]^. Furthermore, bacteria have been detected within tumor cells and tumor-associated immune cells across diverse malignancies, establishing intratumoral microbiota as a bona fide component of the tumor microenvironment^[9]^.

Current research has thus shifted from descriptive evidence to mechanistic and functional interrogation. Intratumoral microbes modulate tumor progression through regulation of inflammation, immune remodeling, metabolic reprogramming, and therapeutic response. Microbial composition correlates with immune infiltration, inflammatory signaling, and treatment sensitivity, while certain bacteria can directly metabolize anticancer drugs or alter host signaling pathways, thereby shaping the efficacy of chemotherapy and immunotherapy^[9]^. Emerging technologies, including single-cell and spatial omics, are further refining our understanding of their spatial organization and host interactions.

Collectively, these findings support a paradigm shift from a tumor cell-centric view to a multidimensional framework integrating tumor-host-microbe interactions. Compared with previous reviews that have primarily summarized the presence, composition, and general biological functions of intratumoral microbiota, this review places greater emphasis on the context dependence and translational limitations of microbial effects. We specifically integrate the routes of microbial entry with tumor-specific ecological niches, examine how spatial localization and host immune status may determine whether microorganisms exert tumor-promoting or antitumor effects, and highlight the crosstalk among microbial-responsive signaling pathways rather than considering these pathways in isolation. We further evaluate the evidence linking intratumoral microbiota to therapeutic responses and distinguish observations supported by mechanistic or preclinical evidence from those that remain largely correlative or clinically unvalidated. Finally, we discuss key technical barriers, including low-biomass contamination, spatial localization, viability assessment, and quantitative analysis, together with practical strategies for improving experimental reliability and clinical translation. Accordingly, this review aims not only to summarize current knowledge but also to identify areas of consensus, unresolved controversies, and evidence gaps that should guide future mechanistic and translational studies.

## SOURCES OF INTRATUMORAL MICROORGANISMS

Intratumoral microorganisms may reach tumor tissues through three major routes: direct invasion from adjacent tissues, hematogenous dissemination, and co-migration with tumor cells during metastasis [Figure 1].

**Figure 1:**
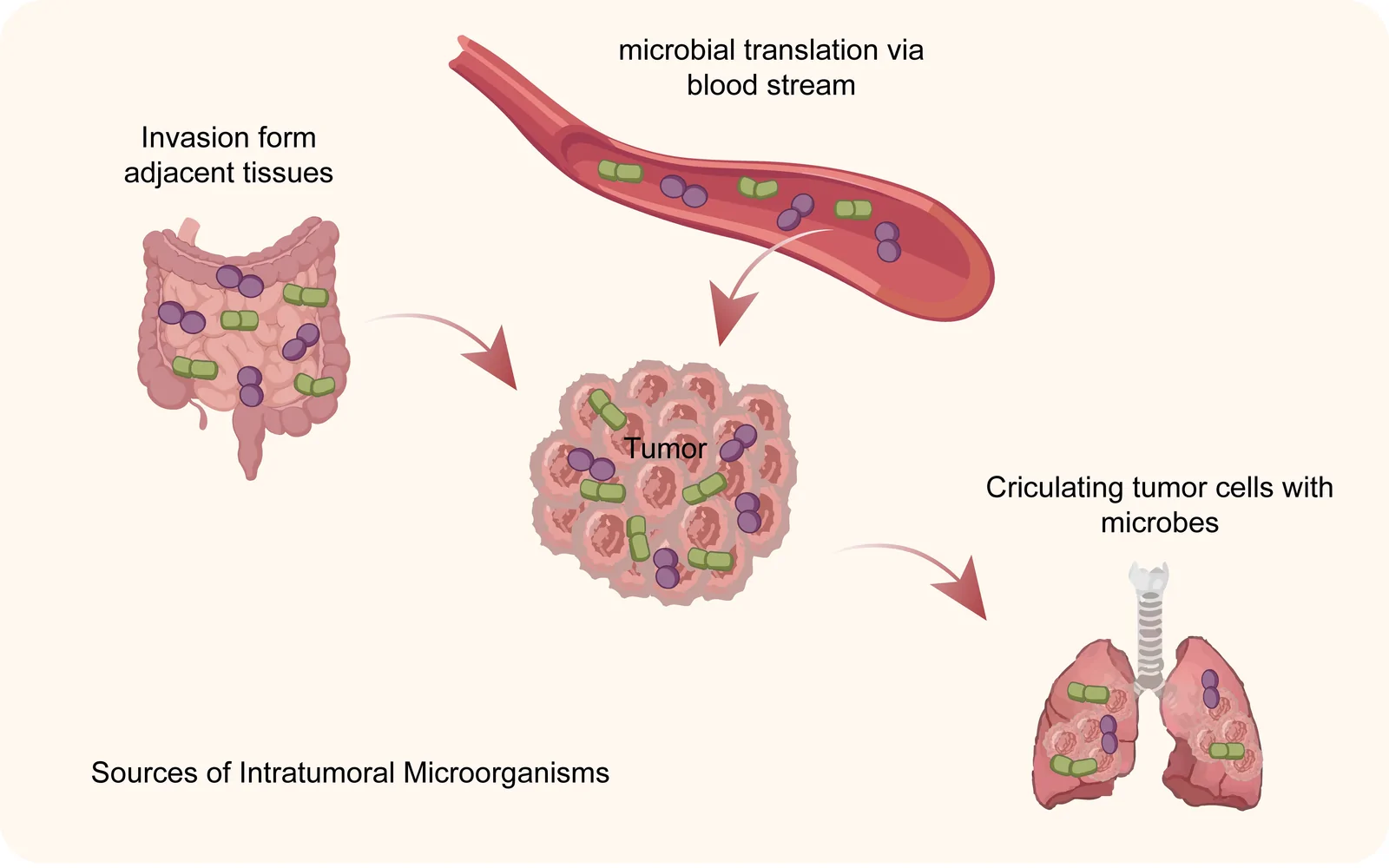
Several sources of microorganisms within tumors. Intratumoral microorganisms can originate from adjacent tissues, bloodstream dissemination, and circulating tumor cells carrying microbes, contributing to microbial colonization within tumor tissues.

### Invasion of microorganisms from adjacent tissues

Among the various pathways for microbial colonization within tumors, the invasion of microorganisms from adjacent tissues is considered a significant source, particularly in tumors occurring in organs that are open to the external environment or inherently rich in commensal microbiota. Sites such as the gastrointestinal tract, respiratory tract, and urogenital tract harbor rich and stable microbial communities^[11,12]^. When tumor growth leads to local tissue destruction, damage to the epithelial barrier, or increased mucosal permeability, microorganisms originally confined to the lumens or surfaces can breach physical barriers and enter tumor tissue^[13,14]^. Tumor-associated chronic inflammatory responses and local hypoxic environments further create conditions conducive to microbial invasion and colonization^[11,14]^. Inflammation can disrupt tight junctions, alter the composition of the mucus layer, and weaken local immune clearance capabilities, while the abnormal metabolic state and immunosuppressive microenvironment of tumor tissue facilitate the long-term survival of certain microorganisms locally and the formation of relatively stable communities.

This phenomenon has been observed in various tumor types. For example, in colorectal cancer tissue, *Fusobacterium nucleatum* of intestinal origin can accumulate within the tumor, promoting tumor cell proliferation via the FadA adhesion molecule while simultaneously suppressing local antitumor immune responses^[15,16]^; In pancreatic ductal adenocarcinoma, members of the **Gammaproteobacteria class**, a group within the phylum **Proteobacteria**, have been detected colonizing tumor tissues and influencing the efficacy of gemcitabine chemotherapy through drug metabolism^[8]^; In lung cancer, Porphyromonas gingivalis of oral origin has been found to invade tumor tissues and participate in tumor progression by regulating inflammatory signaling^[17,18]^. These findings indicate that microorganisms can colonize tumor tissues or interact with tumor-associated immune cells in a cancer type-dependent manner, thereby influencing tumor progression through mechanisms such as modulation of inflammatory responses, immune evasion, and altered tumor cell behavior. However, the underlying mechanisms have primarily been characterized in specific tumor types, and whether they are broadly applicable across different cancers requires further investigation. Overall, the invasion of microorganisms from adjacent tissues provides an important route for the establishment of intratumoral microbiota and highlights the potential link between local microbiome dysbiosis and the tumor microenvironment.

### Hematogenous transmission

Hematogenous spread is considered another major source of intratumoral microbiota, particularly aiding in explaining the presence of microorganisms in solid tumors distant from body cavities or the external environment. Under physiological or pathological conditions, microorganisms and their components can enter the bloodstream via various routes, leading to transient or persistent bacteremia. For example, compromised intestinal barriers, chronic inflammation, invasive medical procedures, and oral mucosal lesions can all facilitate the entry of microorganisms or their metabolites into the circulatory system^[16,19]^. The unique vascular abnormalities and microenvironmental characteristics of tumor tissue create conditions conducive to the retention and colonization of these hematogenous microorganisms. Disrupted tumor vascular architecture, increased vascular permeability, and abnormal hemodynamics make it easier for circulating microorganisms to extravasate and remain at the tumor site^[20,21]^. Concurrently, the pervasive immunosuppressive state of the tumor microenvironment - such as impaired effector immune cell function and reduced phagocytic activity - weakens the host’s ability to clear foreign microorganisms, thereby facilitating their survival and accumulation within tumor tissues^[11,20,22]^.

Related studies provide indirect support across various tumor types. For example, microbial profiles highly similar to those of gut Proteobacteria have been detected in pancreatic ductal adenocarcinoma, suggesting they may migrate from the gut to the tumor site via the circulatory system^[11,22]^; microbial populations such as *Streptococcus* and *Lactobacillus* observed in breast cancer tissue are also considered likely to have originated from the bloodstream^[23,24]^; in melanoma, bacterial signals of oral origin have also been detected in tumor tissues^[25]^. Furthermore, evidence suggests that certain microorganisms can be “carried” to tumor sites after being phagocytosed by circulating immune cells, entering tumor-associated immune cells or tumor cells themselves to achieve cross-tissue transmission^[24,26]^. Overall, the hematogenous route provides a reasonable biological explanation for the formation of intratumoral microbiota, revealing a close connection between the systemic microbiome and the local tumor microenvironment.

### Co-metastasis with tumor cells

In addition to invasion of adjacent tissues and hematogenous spread, intratumoral microbes may also migrate alongside tumor cells during tumor progression and participate in the construction of the microenvironment at metastatic sites. Recent studies have shown that certain microbes can colonize tumor tissues long-term and even enter tumor cells or tumor-associated immune cells, thereby being “carried” to new anatomical sites during tumor cell invasion and distant dissemination^[24,27]^. For example, in breast cancer models, it has been found that bacteria present within tumor cells can help these cells resist oxidative stress during circulation, significantly increasing their survival rate in the bloodstream and promoting distant metastasis^[28]^. During metastasis, circulating tumor cells spread to distant organs via the blood or lymphatic systems, while the coexisting microorganisms may evade immune clearance by remaining intracellular or tightly bound to the cells, arriving at the metastatic site alongside the tumor cells^[16,29]^.

Related studies also suggest that the microbial composition in metastatic lesions bears some resemblance to that of the primary tumor, supporting the possibility that microbes co-metastasize with tumor cells. For instance, *Fusobacterium nucleatum*, which shows high consistency with the primary tumor, has been detected in liver metastases of colorectal cancer^[30]^. It has been confirmed that this bacterium can persist in metastatic lesions and regulate the local immune microenvironment, thereby promoting the growth of metastatic lesions^[30,31]^. These microorganisms may participate in the formation and expansion of metastatic lesions by modulating local immune responses, enhancing tumor cell survival, and promoting colonization at the metastatic site. Furthermore, interactions between tumor cells and microorganisms may confer greater environmental adaptability to tumor cells, such as enhancing their tolerance to stressful conditions or regulating inflammatory signaling pathways, thereby improving their survival rates in the circulation and in the heterogeneous microenvironments of target organs^[29]^. In summary, the co-migration of microorganisms with tumor cells provides a plausible explanation for the continuity of intratumoral microbiota between primary and metastatic sites, suggesting that they may play a significant regulatory role in tumor metastasis and disease progression [Table 1].

**Table 1:** Potential routes of microbial entry into tumor tissues across different cancer types

**Route of microbial entry**	**Representative cancer types**	**Representative microorganisms**	**Evidence and rationale**
Direct invasion from adjacent tissues	Colorectal cancer	*Fusobacterium nucleatum*	Intestinal proximity; tumor colonization^[6,7]^
Hematogenous transmission	Breast cancer; pancreatic ductal adenocarcinoma	*Streptococcus*, *Lactobacillus*; gut-associated Proteobacteria	Bloodstream dissemination; microbial similarity to gut/oral microbiota^[8,32,33]^
Co-metastasis with tumor cells	Metastatic colorectal cancer	*Fusobacterium nucleatum*	Detection in liver metastases; persistence during metastasis^[31,34]^

## FACTORS PROMOTING MICROBIAL COLONIZATION IN THE TUMOR MICROENVIRONMENT

The tumor microenvironment is a complex local ecosystem. Its typical characteristics include persistent hypoxia and metabolic reprogramming, widespread immune suppression and immune evasion, as well as alterations such as abnormal tumor vasculature and increased tissue permeability. These factors collectively shape a niche conducive to microbial survival and accumulation, and largely determine the ability of microorganisms to colonize tumor tissues [Figure 2].

**Figure 2:**
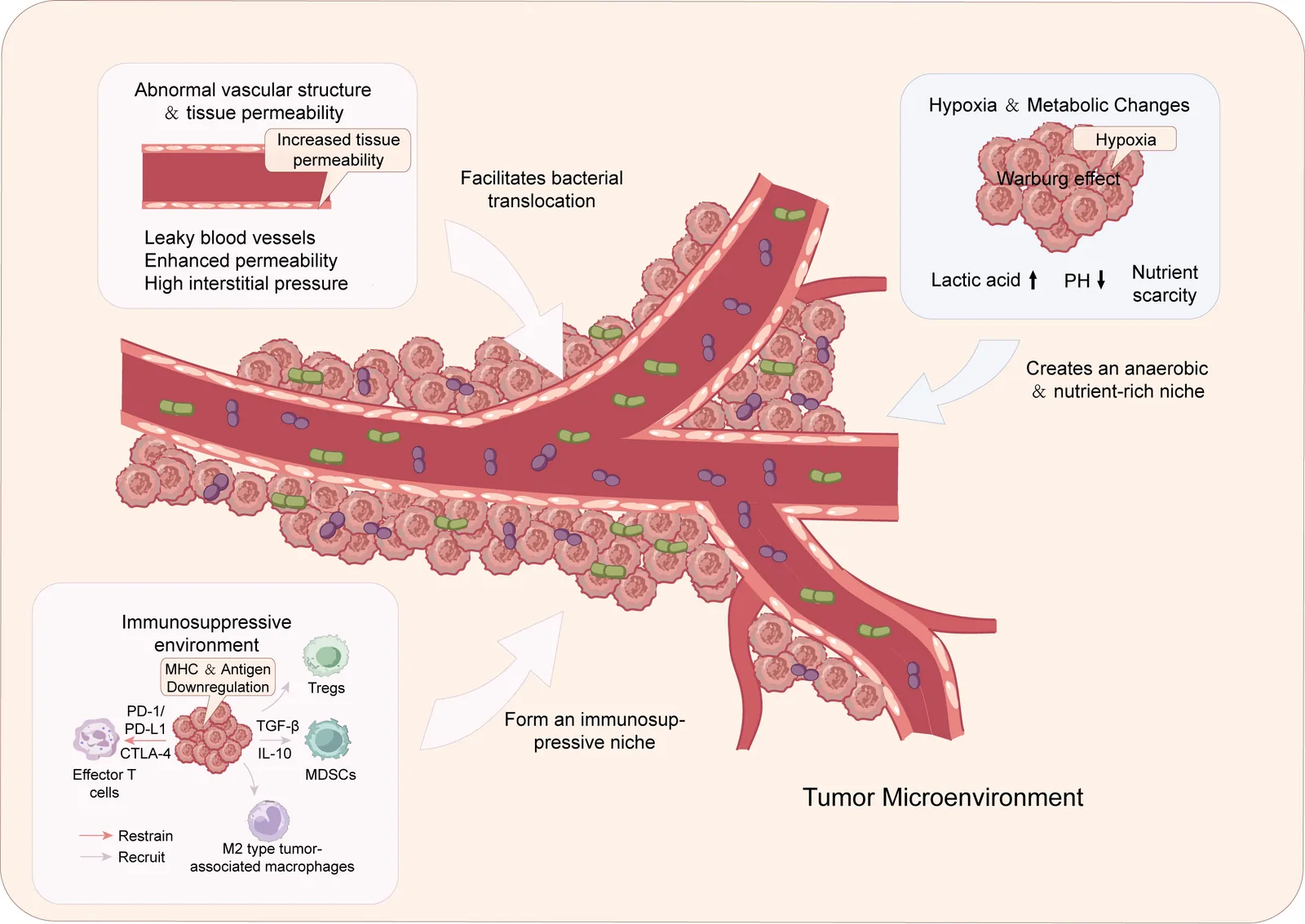
The tumor microenvironment promotes microbial colonization. The tumor microenvironment promotes microbial accumulation through abnormal vascular permeability, hypoxia-induced metabolic changes, and immunosuppressive conditions. These factors create a favorable niche for microbial survival and colonization within tumors. MHC: Major histocompatibility complex; PD-1: programmed cell death protein 1; PD-L1: programmed death-ligand 1; CTLA-4: cytotoxic T-lymphocyte-associated protein 4; MDSC: Myeloid-derived suppressor cell; Tregs: regulatory T cells; TGF-β: transforming growth factor beta; IL-6: interleukin-6; IL-10: interleukin-10; M2: alternatively activated macrophages.

### Hypoxic environment and metabolic changes

During the growth of solid tumors, metabolic reprogramming characterized by enhanced glycolysis and lactate accumulation, together with abnormal angiogenesis and heterogeneous blood perfusion, creates persistent hypoxic and metabolically heterogeneous niches within the tumor microenvironment^[35,36]^.

Hypoxic or even near-anoxic environments favor the survival of anaerobic and facultative anaerobic bacteria, while accumulated lactate and other locally available metabolites may provide carbon sources or signaling cues for some tumor-associated microorganisms^[37,38]^.

Previos studies have shown that Fusobacterium nucleatum, which is enriched in colorectal cancer tissues, can adapt to hypoxic and high-lactate environments, with metabolic characteristics that may confer a survival advantage in the tumor core compared with normal mucosa^[39]^. Similarly, anaerobic or facultative anaerobic bacteria detected in hypoxic pancreatic ductal adenocarcinomas have been associated with the tumor-specific metabolic environment^[40]^. These findings suggest that tumor-associated hypoxia and metabolic reprogramming are not only key drivers of tumor progression but may also participate in the dynamic regulation of the tumor microenvironment by shaping specific niches that promote the selective colonization and persistence of particular microorganisms within solid tumors.

### Immunosuppression and immune evasion

During tumor initiation and progression, tumor cells gradually shape a microenvironment characterized by immune suppression and immune evasion through multifaceted mechanisms. On the one hand, tumor cells can downregulate the expression of major histocompatibility complex (MHC) molecules and tumor-associated antigens, thereby reducing their immunogenicity and impairing the recognition capacity of effector T cells^[41,42]^. On the other hand, they inhibit T-cell activation and effector functions by activating immune checkpoint pathways such as programmed cell death protein 1/programmed death-ligand 1 (PD-1/PD-L1) and cytotoxic T-lymphocyte-associated protein 4 (CTLA-4), thereby suppressing T-cell activation and effector functions^[43,44]^. Furthermore, tumor cells and their stromal components secrete immunosuppressive factors such as transforming growth factor beta (TGF-β) and interleukin-10 (IL-10), and recruit regulatory T cells (Tregs), myeloid-derived suppressor cells (MDSCs), and M2-type tumor-associated macrophages, further attenuating antitumor immune responses^[42,45]^. Concurrently, metabolic stresses - such as hypoxia, acidification, and competition for nutrients - induced by rapid tumor growth can lead to functional exhaustion and metabolic dysfunction in effector T cells, collectively forming a crucial biological basis for tumor immune evasion^[44,46]^.

The immunosuppressive tumor microenvironment also provides a permissive niche for the persistence of intratumoral microorganisms. Impaired antigen presentation, reduced effector immune-cell function, and increased immunoregulatory activity may facilitate microbial survival and long-term colonization within tumor tissues. Conversely, intratumoral microorganisms can further modify local immune and inflammatory states, creating reciprocal interactions between microbial persistence and tumor immune suppression.

### Abnormal vascular architecture and tissue permeability

Tumor-associated angiogenesis is characterized by abnormal vessel architecture and increased vascular permeability, driven by persistent activation of pro-angiogenic signals such as vascular endothelial growth factor (VEGF), fibroblast growth factor (FGF), and platelet-derived growth factor (PDGF)^[47]^. These abnormal vessels exhibit impaired endothelial junctions and barrier function, resulting in increased vascular permeability and leakage^[48]^. These abnormalities increase vascular leakage and interstitial pressure, contributing to spatially heterogeneous tumor microenvironments^[48]^. Importantly, increased vascular permeability may also facilitate the entry of circulating microorganisms or microbial components into tumor tissues.

Recent studies indicate that abnormal vascular and tissue permeability constitute a critical microenvironmental foundation for microbial colonization within tumors. For example, in pancreatic ductal adenocarcinoma and breast cancer, metagenomic sequencing combined with *in situ* hybridization revealed that low-abundance but stable bacterial signals frequently cluster in areas with abundant and structurally abnormal vasculature^[49,50]^; in colorectal cancer and melanoma models, experimentally enhancing vascular permeability increases microbial enrichment within tumors, whereas inhibiting VEGF signaling and promoting vascular “normalization” partially reduces microbial load^[51,52]^. These findings suggest that the “leakage” of abnormal blood vessels provides favorable conditions for circulating microbes to cross the blood-tumor barrier and colonize tumors.

### Mechanisms of microbial adaptation to the intratumoral ecosystem

Microbial colonization in the tumor microenvironment is not a passive, unidirectional process; rather, microorganisms gradually integrate into the tumor ecosystem through a series of active adaptation and regulatory mechanisms. Existing research indicates that intratumoral microorganisms achieve long-term survival and stable colonization through various means, including metabolic reprogramming, immune regulation, and spatial remodeling of the microenvironment^[25]^.

At the metabolic level, lactic acid accumulation, amino acid imbalance, and lipid metabolism abnormalities are prevalent in tumor tissues; some microorganisms can utilize these unconventional metabolic substrates to sustain growth. For example, anaerobic bacteria such as *Bacteroides* and *Peptoniphilus*, detected in pancreatic ductal adenocarcinoma, can adapt to a hypoxic, high-lactate metabolic environment and are associated with tumor immunosuppression^[53]^. Additionally, tumor-associated microorganisms produce short-chain fatty acids, polyamines, or purine metabolites. In terms of immune regulation, some tumor-associated microorganisms suppress antitumor immune responses, enabling the tumor to evade immune clearance. For example, *Streptococcus anginosus,* which is enriched in gastric cancer tissues, has been reported to influence macrophage polarization and inflammatory signaling pathways, promoting the formation of an immunosuppressive microenvironment^[54,55]^. Furthermore, intratumoral microbiota can induce the expression of immunosuppressive cytokines such as IL-10 and TGF-β, or upregulate immune checkpoint molecules like PD-L1, thereby impairing the effector functions of CD8^+^ T cells and natural killer cells^[26,38]^. Furthermore, certain microorganisms can alter local tissue architecture by regulating extracellular matrix remodeling or inducing chronic low-grade inflammation, thereby creating more favorable spatial conditions for their own colonization^[38]^. These adaptive mechanisms enable microorganisms to survive long-term in the tumor microenvironment - characterized by nutritional abnormalities, immunosuppression, and structural disruption - and to form a mutually reinforcing functional symbiotic relationship with the tumor.

Together, these adaptive processes allow intratumoral microorganisms to persist under the metabolic, immune, and structural constraints of tumor tissues. The resulting ecological interactions provide a basis for understanding their heterogeneous distribution and context-dependent biological effects.

## COMPOSITION AND SPATIAL DISTRIBUTION OF INTRATUMORAL MICROBIOTA

Intratumoral microbiota exhibit both common features and significant specificity across different cancer types. Extremely low levels of microbial signals can be detected in most solid tumors, primarily consisting of anaerobic or facultative anaerobic bacteria. However, there are often marked differences in microbial composition between different cancer types, which may be related to tissue origin, local microenvironmental characteristics, and host factors. Furthermore, intratumoral microbiota are not uniformly distributed but exhibit significant spatial heterogeneity. Microbes tend to accumulate in necrotic areas, hypoxic zones, regions with vascular abnormalities, and areas of immune cell infiltration. Some may be localized within tumor cells or tumor-associated immune cells, while others are primarily distributed in the stroma or extracellular matrix. Microbial composition may also vary between different regions within the same tumor (e.g., the tumor core versus the invasive front, or the primary tumor versus metastatic sites). The functional consequences of intratumoral microbiota may also depend on their spatial localization within tumors. Microbes enriched in the hypoxic and necrotic tumor core may adapt to metabolic stress and contribute to immune suppression or reduced sensitivity to oxygen-dependent therapies. In contrast, microorganisms located at the invasive front may interact more directly with tumor cells, extracellular matrix, and infiltrating immune cells, potentially influencing tumor cell migration, invasion, and immune surveillance. Microbes localized near blood vessels or immune-cell-rich regions may further affect immune-cell recruitment and activation, thereby altering local immune evasion and treatment responses. These spatially dependent effects suggest that microbial function cannot be fully understood from bulk microbial abundance alone and should be interpreted together with the local metabolic, structural, and immune context.

### Extremely low microbial biomass and the dominance of facultative anaerobes

In the field of intratumoral microbiology, “extremely low biomass” is considered one of its most fundamental ecological characteristics and has long been a major source of academic controversy. Compared with high-biomass microbial niches such as the gut, oral cavity, and skin, microbial signals in tumors are relatively scarce and highly susceptible to technical contamination^[56]^.

Multi-cancer studies using *in situ* hybridization, electron microscopy, and contamination controls have confirmed the presence of bacteria in various solid tumor tissues^[9]^. Their abundance was substantially lower than that of classical microbial ecosystems, and many were detected intracellularly within tumor cells or tumor-associated immune cells^[9,57]^. Tumor-associated hypoxia and fluctuating oxygen gradients may favor facultative anaerobes, which can switch between aerobic and fermentative metabolism and therefore adapt to heterogeneous oxygen availability within tumor tissues^[58]^.

Multi-cancer sequencing studies have identified proteobacteria and firmicutes among the predominant phyla in tumor tissues^[9,56]^. In pancreatic ductal adenocarcinoma, tumor-enriched γ-proteobacteria have been reported to express cytidine deaminase and inactivate gemcitabine^[8]^. Multi-cancer sequencing studies have identified Proteobacteria and Firmicutes among the predominant phyla in tumor tissues^[9,56]^. In pancreatic ductal adenocarcinoma, tumor-enriched γ-proteobacteria have been reported to express cytidine deaminase and inactivate gemcitabine [8], illustrating that microbial metabolic traits may contribute to adaptation within the tumor microenvironment.

In colorectal cancer, facultative anaerobes such as Escherichia coli have also been detected and may exploit tumor-associated metabolites under hypoxic conditions^[59,60]^. In pancreatic cancer, differences in Proteobacteria-dominated microbial profiles have been associated with patient survival, suggesting that microbial composition may reflect tumor-specific ecological conditions^[10]^. Overall, the combination of low microbial biomass and relative enrichment of facultative anaerobes reflects the selective influence of oxygen availability and other microenvironmental conditions on intratumoral microbial communities^[61]^.

### Significant tumor-type specificity and spatial heterogeneity of intratumoral microbiota

Intratumoral microbiota exhibit marked tumor-type specificity, with distinct microbial profiles reported across different cancers. For example, breast cancer tissues are enriched in Proteobacteria and Methylobacterium, pancreatic ductal adenocarcinomas in γ-Proteobacteria, and lung tumors in oral-associated genera such as Veillonella and Prevotella^[9,62]^; Microbial communities in melanoma and osteosarcoma also differ from those observed in gastrointestinal tumors^[63]^. These differences may reflect variation in tissue origin, metabolism, oxygenation, and immune context, suggesting that local tumor niches influence microbial composition.

Intratumoral microbiota also exhibit substantial spatial heterogeneity, with microorganisms preferentially localized to distinct tumor compartments^[64,65]^. Microorganisms may preferentially localize to hypoxic or necrotic regions, areas with abnormal vasculature, or immune-cell-rich compartments, and can occur within tumor cells or tumor-associated immune cells^[26,64]^.

Microbial composition may also differ between primary and metastatic sites, reflecting both retention of tumor-associated microbial features and adaptation to the local metabolic, oxygen, and immune environment^[29,66]^. This cross-organ microecological adjustment suggests that the tumor-associated microbial community possesses a certain degree of dynamic plasticity rather than being a static accompanying phenomenon.

## FUNCTIONAL ROLES OF INTRATUMORAL MICROBIOTA IN TUMOR INITIATION AND PROGRESSION

Intratumoral microbiota can influence tumor initiation and progression through direct interactions with tumor cells and indirect effects on the immune and metabolic microenvironment. However, these effects are not uniformly tumor-promoting or tumor-suppressive and may vary according to microbial composition, spatial localization, metabolic conditions, and host immune status. The following sections summarize the major tumor-promoting and tumor-suppressive mechanisms, with particular attention to the context dependence of these effects and the limitations of current evidence.

### Tumor-promoting mechanisms

Tumor-promoting effects can be broadly categorized into oncogenic signaling, inflammatory and immune remodeling, metabolic regulation, stress adaptation, and genomic instability [Figure 3].

**Figure 3:**
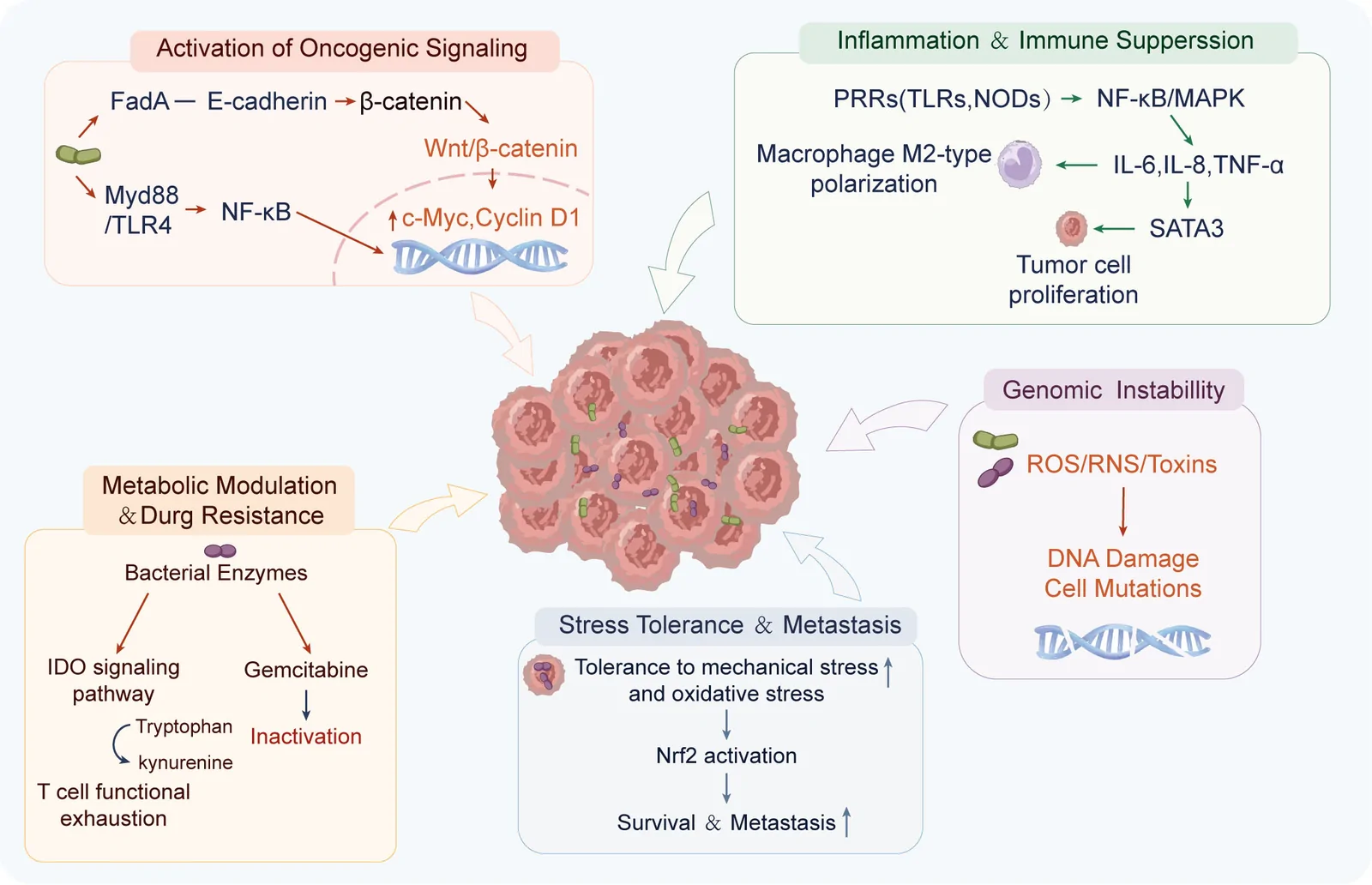
The mechanism by which microorganisms within tumors promote tumor growth. Intratumoral microorganisms contribute to tumor development through activation of oncogenic signaling, induction of inflammation and immune suppression, metabolic reprogramming and drug resistance, genomic instability, and enhancement of stress tolerance and metastasis. FadA: Fusobacterium adhesin A; TLR: Toll-like receptor; MyD88: myeloid differentiation primary response 88; NOD: nucleotide-binding oligomerization domain-containing protein; NF-κB: nuclear factor kappa-B; MAPK: mitogen-activated protein kinase; IL-6: interleukin-6; IL-8: interleukin-8; TNF-α: tumor necrosis factor alpha; STAT3: signal transducer and activator of transcription 3; PI3K: phosphoinositide 3-kinase; Akt: protein kinase B; mTOR: mechanistic target of rapamycin; ROS: reactive oxygen species; RNS: reactive nitrogen species; Nrf2: nuclear factor erythroid 2-related factor 2; IDO: indoleamine 2,3-dioxygenase.

(1) Direct activation of oncogenic signaling pathways to promote tumor cell proliferation

In colorectal cancer, *Fusobacterium nucleatum* is considered a typical representative. Its surface adhesion molecule FadA binds to E-cadherin on the surface of tumor cells, inducing the dissociation of β-catenin from the cell membrane and its translocation into the nucleus. This activates the Wnt/β-catenin signaling pathway, upregulating the expression of pro-proliferative genes such as c-Myc and Cyclin D1, thereby promoting cell cycle progression and tumor proliferation^[67,68]^. Furthermore, this bacterium can further enhance NF-κB activation by activating TLR4/MyD88-dependent signaling, inducing the expression of inflammation-related and anti-apoptotic genes, and creating a microenvironment characterized by both proliferation and chronic inflammation^[67,69]^. In oral squamous cell carcinoma and esophageal cancer, *Porphyromonas gingivalis* can invade tumor cells and survive long-term. Its infection activates the phosphoinositide 3-kinase/protein kinase B (PI3K/Akt) and Janus kinase/signal transducer and activator of transcription (JAK/STAT) signaling pathways, inhibits caspase-dependent apoptosis, and simultaneously enhances the expression of molecules such as Cyclin D1 and Bcl-2, thereby promoting cell cycle progression and increasing cell survival rates^[70]^.

(2) Induction of chronic inflammation and an immunosuppressive microenvironment

Intratumoral microbiota can drive a state of chronic, low-grade inflammation by continuously activating pattern recognition receptors (PRRs), including Toll-like receptors (TLRs) and NOD-like receptors. For example, in gastric cancer, in addition to *Helicobacter pylori*, oral-derived bacterial communities such as *Streptococcus anginosus* accumulate in tumor tissues and have been reported to activate NF-κB and MAPK signaling pathways, inducing the expression of pro-inflammatory factors such as IL-6, IL-8, and TNF-α^[55,71]^. These cytokines promote tumor cell proliferation via the STAT3 pathway while driving macrophage polarization toward the M2 phenotype, thereby creating an immunosuppressive microenvironment^[71]^. In colorectal cancer, the Fap2 protein from *F. nucleatum* binds to TIGIT on the surface of immune cells, inhibiting the cytotoxic function of CD8^+^ T cells and NK cells; simultaneously, by inducing the expression of chemokines such as CCL20, it promotes the infiltration of immunosuppressive cells, thereby weakening antitumor immune clearance^[50,72]^. Consequently, microorganisms enhance tumor cell proliferation through intracellular signaling while reducing the host’s clearance capacity through immune regulation.

(3) Regulating metabolism and treatment response to indirectly promote sustained tumor growth

In pancreatic ductal adenocarcinoma, Gammaproteobacteria enriched within the tumor can express bacterial cytidine deaminase, which metabolizes the chemotherapeutic drug gemcitabine into inactive metabolites, thereby reducing treatment efficacy and indirectly promoting sustained tumor proliferation^[8,73]^. In addition to drug metabolism, microorganisms can influence tumor cell energy metabolism and immune evasion by regulating lactate production, fatty acid oxidation, and tryptophan metabolism^[38]^. For example, microbial metabolites can enhance the activity of indoleamine 2,3-dioxygenase (IDO)-related pathways, promote the conversion of tryptophan to kynurenine, induce T-cell functional exhaustion, and enhance immune tolerance, thereby indirectly sustaining continuous tumor growth^[38]^.

(4) Enhancing stress tolerance and metastatic potential

In breast cancer and melanoma models, *in situ* studies have revealed that certain bacteria can reside intracellularly within tumor cells. Animal studies suggest that these intracellular bacteria can enhance tumor cells’ tolerance to mechanical and oxidative stress, improving the survival of circulating tumor cells under blood flow shear stress and oxidative stress^[24]^. Mechanistically, this may involve enhanced integrin signaling, cytoskeletal remodeling, and activation of antioxidant response pathways (such as nuclear factor erythroid 2-related factor 2), thereby increasing the survival advantage of tumor cells during circulation and distant colonization, and promoting metastasis^[9,24]^.

(5) Induction of genomic instability and DNA damage

Some tumor-associated bacteria may also directly induce double-strand breaks or base damage in DNA by producing reactive oxygen species (ROS), reactive nitrogen species (RNS), or genotoxins (such as colibactin produced by certain *E. coli* strains), thereby increasing the mutational burden and chromosomal instability, and providing a genetic basis for tumor evolution^[74,75]^.

### Antitumor mechanisms

Although intratumoral microbiota are associated with tumor progression in most contexts, growing evidence suggests that, under specific conditions regarding microbial composition, spatial localization, and host immune status, certain tumor-associated microorganisms can exert antitumor effects through various mechanisms, including enhancing antitumor immunity, inducing tumor cell death, disrupting tumor metabolism, and regulating vascular and inflammatory homeostasis. This context-dependent effect reflects the highly dynamic equilibrium of the intratumoral microbiota. A conceptual microbial-host balance model may help explain these context-dependent effects. The net impact of intratumoral microbiota is likely determined by the combined effects of microbial composition, abundance, spatial localization, and host immune status. Microbial communities enriched in tumor-promoting species, present at sufficient abundance, or localized within tumor cells or immunosuppressive niches may favor chronic inflammation, immune evasion, metabolic adaptation, and tumor progression. In contrast, microbial communities or metabolites that enhance antigen presentation, activate cyclic GMP-AMP synthase-stimulator of interferon genes (cGAS-STING) signaling, promote antitumor immune polarization, or induce tumor cell stress may shift the balance toward tumor suppression. Importantly, microbial abundance alone is unlikely to determine the biological outcome; the functional properties of individual microbial taxa, their spatial distribution, and the immune context of the tumor may be equally or more important. Thus, the effects of intratumoral microbiota should be considered as a dynamic balance between tumor-promoting and tumor-suppressive signals rather than as a uniform property of the microbiome.

(1) Enhancement of antigen immunogenicity and CD8^+^ T cell responses

Intratumoral microbes can increase the “immunological visibility” of tumor cells. In melanoma models, studies have found that certain peptides derived from intracellular bacteria can be loaded onto MHC-I molecules and cross-presented to CD8^+^ T cells by tumor cells or dendritic cells, thereby enhancing specific cytotoxic responses. This mechanism of “microbial-associated antigen cross-presentation” enhances the immunogenicity of tumor antigens, promotes IFN-γ secretion and the release of cytotoxic molecules (such as granzyme B), and improves tumor clearance efficiency^[76,77]^. In pancreatic ductal adenocarcinoma, intratumoral microbiota have been associated with tumor immunity and clinical outcomes^[53]^. Experimental studies further indicate that the pancreatic cancer microbiome can regulate innate and adaptive immune responses through TLR-dependent mechanisms, including effects on myeloid-cell differentiation and T-cell function^[78,79]^. Conversely, following treatment with broad-spectrum antibiotics, CD8^+^ T cell infiltration decreases, IFN-γ levels drop, and tumor growth accelerates, suggesting that a certain degree of microbial stimulation supports the maintenance of antitumor immunity^[8,10]^. However, because systemic antibiotic treatment affects both the gut and intratumoral microbiota, the relative contribution of intratumoral microorganisms to these immune alterations remains to be fully elucidated.

(2) Activation of the cGAS-STING-type I interferon pathway

Bacterial DNA within tumors can be recognized by the host cell’s DNA sensor cGAS, thereby activating the STING signaling pathway and inducing type I interferon production, while bacteriogenic cyclic dinucleotides can directly activate STING^[80]^. Type I interferons play a central role in antitumor immunity, with functions including: promoting cross-presentation by dendritic cells, enhancing CD8^+^ T cell expansion and memory formation, and inhibiting tumor angiogenesis^[81]^. In certain melanoma and colorectal cancer models, microbiome-induced STING activation is associated with tumor growth suppression, suggesting that moderate stimulation of the innate immune system can be converted into an effective adaptive immune response^[81]^.

(3) Regulation of immune cell polarization and inflammatory balance

Different microorganisms can regulate the differentiation of immune cells through pattern recognition receptors (such as TLR2, TLR5, and TLR9). Certain microbial communities can promote the polarization of macrophages toward the M1 phenotype, enhancing iNOS and TNF-α expression and increasing tumor-killing capacity^[82,83]^. Concurrently, the enhancement of Th1-type immune responses (increased IL-12 and IFN-γ) can suppress Treg expansion and reduce the proportion of M2-type macrophages, thereby improving the immunosuppressive microenvironment^[83]^. Experimental models demonstrate that stimulation by specific anaerobic bacteria can enhance the expression of Th1-associated cytokines and boost CD8^+^ T cell function, creating an inflammatory state more conducive to tumor clearance^[83,84]^.

(4) Induction of tumor cell apoptosis and cell cycle arrest

For example, gut microbiota-derived butyrate, a short-chain fatty acid, has been shown to inhibit histone deacetylase (HDAC) activity, leading to chromatin opening and enhanced expression of tumor suppressor genes^[85,86]^. This process promotes the expression of cell cycle inhibitors such as p21 and p27, activates the Bax-caspase-dependent apoptotic pathway, and suppresses Wnt/β-catenin signaling^[87]^. Although these findings highlight the important role of microbiota-derived metabolites in the epigenetic regulation of tumor cells, the available evidence is derived mainly from studies of the gut microbiota, and whether intratumoral microbiota exert similar effects remains to be determined.

### Regulation of antitumor immunity by intratumoral microorganisms

The regulation of antitumor immunity by intratumoral microbiota is not limited to intervention at a single level but involves multi-tiered mechanisms. These include interfering with antigen recognition, suppressing effector cell function, reshaping myeloid cell differentiation, inducing immune checkpoint expression, and maintaining chronic inflammation-driven immune suppression networks, thereby systematically weakening the host’s immune surveillance and clearance capabilities [Figure 4].

**Figure 4:**
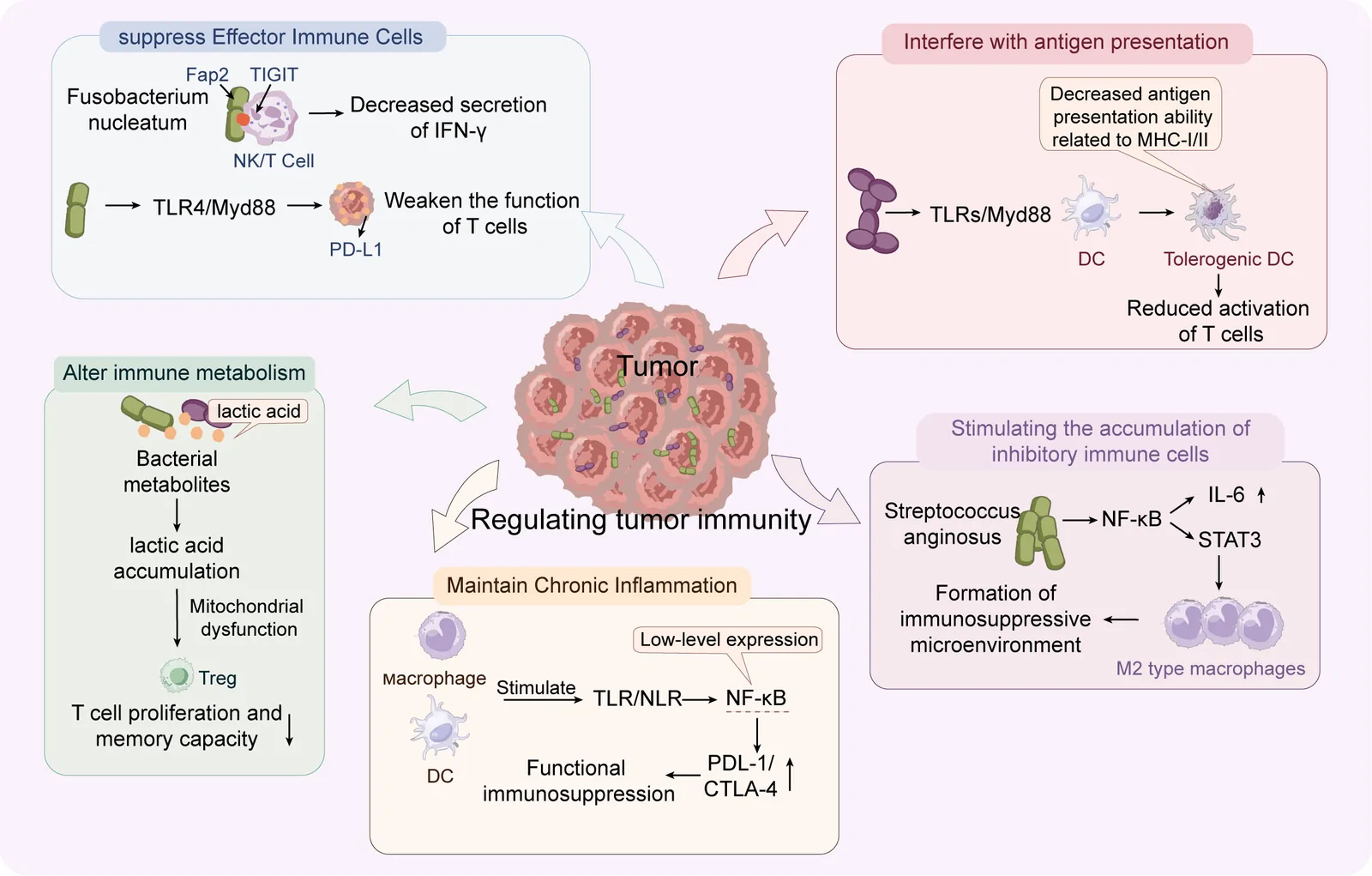
The regulation of tumor immunity by microorganisms within the tumor. Intratumoral microorganisms modulate antitumor immunity by suppressing effector immune cells, impairing antigen presentation, promoting the accumulation of immunosuppressive cells, maintaining chronic inflammation, and altering immune metabolism. Fap2: Fusobacterium adhesin protein 2; TIGIT: T cell immunoreceptor with Ig and ITIM domains; TLR: Toll-like receptors; MyD88: myeloid differentiation primary response 88; NLR: nucleotide-binding domain-like receptor; NF-κB: nuclear factor kappa-B; PD-L1: programmed death-ligand 1; CTLA-4: cytotoxic T-lymphocyte-associated protein 4; DC: dendritic cell; MHC-I/II: major histocompatibility complex class I and class II; Treg: regulatory T cell; IFN-γ: interferon-gamma; IL-6: interleukin-6; STAT3: signal transducer and activator of transcription 3; M2: alternatively activated macrophages.

(1) Direct inhibition of cytotoxic function in effector immune cells

In colorectal cancer, *Fusobacterium nucleatum* is the most extensively studied example. Its outer membrane protein Fap2 binds to the inhibitory receptor TIGIT on the surface of immune cells, directly inhibiting the granule release and IFN-γ secretion of NK cells and CD8^+^ T cells, thereby reducing their cytotoxic activity against tumor cells^[72,88]^. Concurrently, this bacterium can also upregulate PD-L1 expression on tumor cells via the TLR4/MyD88 signaling pathway, enhancing immune suppression mediated by the PD-1/PD-L1 axis and further impairing T-cell function^[89]^.

(2) Interference with antigen presentation and dendritic cell function

Intratumoral microbiota can activate innate immune signaling through pattern recognition receptors (such as TLR2, TLR4, and NOD-like receptors), and dysregulated microbial signaling may contribute to immune tolerance or suppression. In a pancreatic ductal adenocarcinoma model, tumor-associated microbiota have been reported to influence dendritic cell (DC) function through TLR-MyD88-dependent signaling, resulting in reduced expression of co-stimulatory molecules such as CD80/CD86 and impaired antigen-presenting capacity, thereby potentially limiting the effective activation of T cells^[78,90,91]^. Antibiotic-induced depletion of the microbiota or the use of MyD88-deficient models restores DC function and enhances CD8^+^ T cell infiltration, suggesting a critical role for microbiota in suppressing antigen presentation^[92]^. However, because systemic antibiotic treatment affects both the gut and intratumoral microbiota, the specific contribution of intratumoral microorganisms to these immunological changes remains to be further clarified.

(3) Driving the accumulation of immunosuppressive myeloid cells and regulatory T cells

In various solid tumors, intratumoral microbiota can induce the secretion of immunoregulatory factors such as IL-6, IL-10, and TGF-β by activating NF-κB, STAT3, and MAPK pathways. These factors promote the expansion of myeloid-derived suppressor cells (MDSCs) while driving macrophage polarization toward the M2 phenotype^[93]^. For example, *Streptococcus anginosus*, which is enriched in gastric cancer tissues, has been reported to continuously activate NF-κB signaling, enhance IL-6 expression, and activate the STAT3 pathway, thereby increasing the proportion of M2 macrophages and creating an immunosuppressive microenvironment^[54]^. Concurrently, a state of chronic inflammation promotes the recruitment and expansion of regulatory T cells (Tregs), further suppressing the function of effector T cells.

(4) Maintenance of chronic, low-grade inflammation and “functional immune paralysis”

Some tumor-associated bacteria can survive within macrophages or dendritic cells without being completely cleared, resulting in a “phagocytosed but not eliminated” state. This persistence can repeatedly stimulate TLR and NLR signaling, maintaining low-level expression of NF-κB and inflammatory cytokines^[94]^. However, this inflammatory state does not generate an effective cytotoxic response; instead, it is accompanied by the upregulation of immune checkpoint molecules (such as PD-L1 and CTLA-4-related pathways), leading the immune system into a state of chronic activation but with reduced efficacy - that is, “functional immune suppression”^[95]^. In colorectal cancer, *F. nucleatum* can also promote the recruitment of CCR6^+^ immunosuppressive cells by inducing CCL20 expression, exacerbating the coexistence of inflammation and immunosuppression^[95]^. Similar phenomena have been reported in models of liver and pancreatic cancer, suggesting that chronic inflammation-driven immune tolerance is a key regulatory mechanism of the tumor microbiome.

(5) Effects on immune metabolism and T-cell functional state

Intratumoral microbiota and their metabolites can also regulate immune metabolic networks. For example, certain microbial communities can enhance the activity of the tryptophan-kynurenine pathway, indirectly promoting IDO-related immunosuppression^[96]^; lactate accumulation and alterations in lipid metabolism can affect T-cell mitochondrial function and mTOR signaling activity, thereby impairing the proliferation and memory formation capabilities of effector T cells^[96]^. Together, these mechanisms illustrate how intratumoral microbiota can reshape the immune microenvironment and weaken antitumor immune surveillance.

### Regulation of signaling pathways by intratumoral microbiota

The major signaling pathways described above do not operate independently. Rather, microbial stimuli can converge on interconnected host signaling networks that link inflammatory sensing, oncogenic signaling, immune regulation, and metabolic adaptation [Figure 5]. This section therefore focuses on pathway-level interactions and crosstalk [Figure 5].

**Figure 5:**
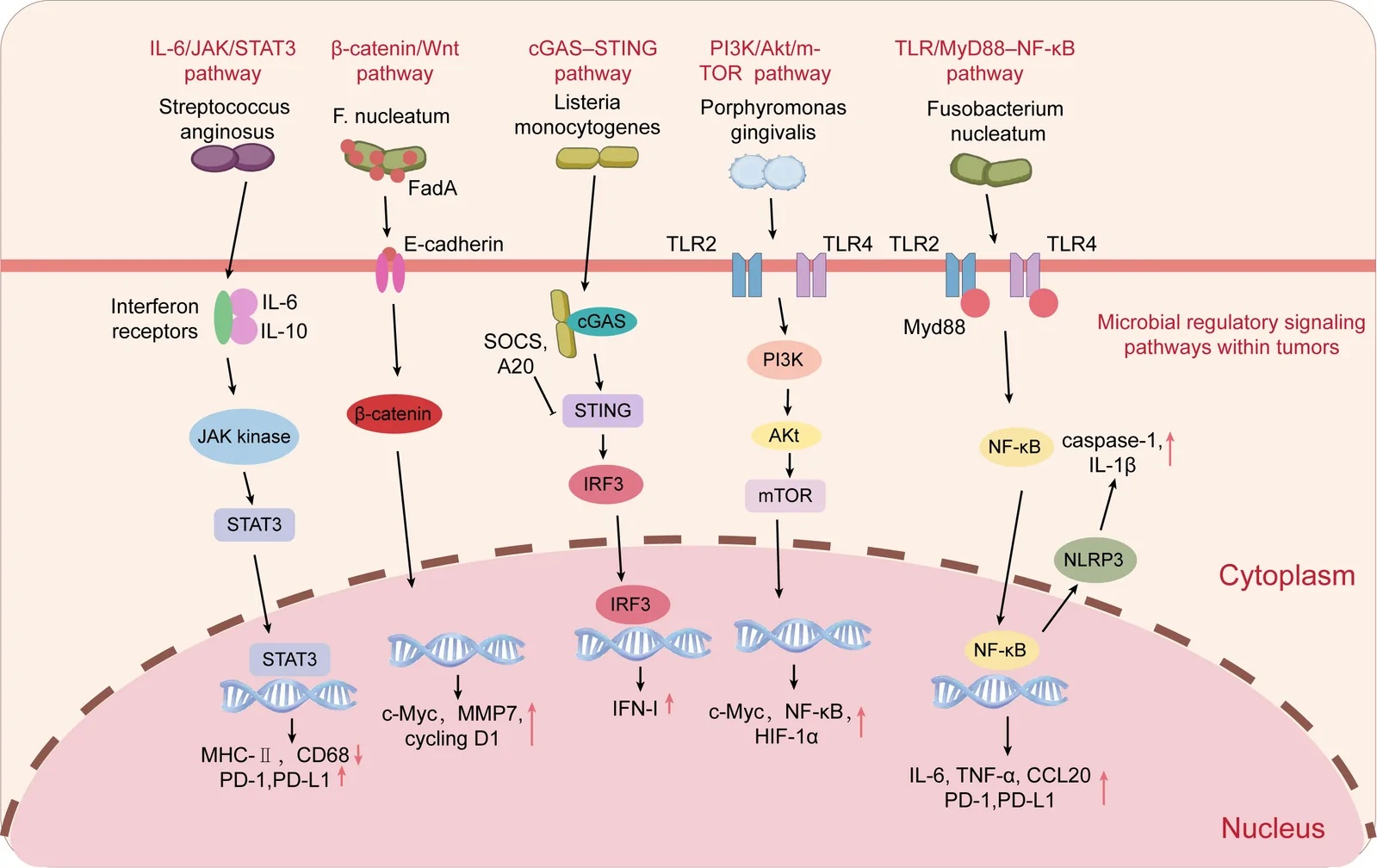
The regulation of signaling pathways by microorganisms within tumors. Intratumoral microorganisms regulate tumor progression and immune responses through multiple host signaling pathways, including IL-6/JAK/STAT3, β-catenin/Wnt, cGAS-STING, PI3K/Akt/mTOR, and TLR/MyD88/NF-κB pathways. IL-6: Interleukin-6; IL-10: interleukin-10; JAK: Janus kinase; STAT3: signal transducer and activator of transcription 3; FadA: Fusobacterium adhesin A; cGAS: cyclic GMP-AMP synthase; STING: stimulator of interferon genes; SOCS: suppressor of cytokine signaling; A20: tumor necrosis factor alpha-induced protein 3; IRF3: interferon regulatory factor 3; PI3K: phosphoinositide 3-kinase; Akt: protein kinase B; mTOR: mechanistic target of rapamycin; TLR: Toll-like receptor; MyD88: myeloid differentiation primary response 88; NF-κB: nuclear factor kappa-B; NLRP3: NOD-like receptor family pyrin domain-containing 3; MMP7: matrix metalloproteinase 7; HIF-1α: hypoxia-inducible factor 1 alpha; ROS: reactive oxygen species; TNF-α: tumor necrosis factor alpha; CCL20: C-C motif chemokine ligand 20; IFN-I: type I interferon; PD-1: programmed cell death protein 1; PD-L1: programmed death-ligand 1.

(1) TLR/MyD88-NF-κB pathway

Microbial activation of TLR2/TLR4-MyD88 signaling induces NF-κB-dependent inflammatory and immunoregulatory programs. In colorectal cancer, F. nucleatum-derived signals can activate this axis and increase IL-6, TNF-α, and CCL20, linking microbial sensing to chronic inflammation and immune suppression^[50]^.

(2) IL-6/JAK/STAT3 pathway

The IL-6/JAK/STAT3 pathway can function downstream of microbial-induced inflammatory signaling. In gastric cancer, Streptococcus anginosus has been reported to induce IL-6 secretion and activate JAK/STAT3 signaling, which is associated with impaired dendritic-cell maturation and increased M2 macrophage and Treg accumulation^[54]^. Thus, NF-κB-driven cytokine production and STAT3 activation may constitute a functional signaling axis linking microbial sensing to persistent immune suppression.

(3) β-catenin/Wnt pathway

The Wnt/β-catenin pathway provides another signaling route through which intratumoral microbes may influence tumor behavior and immune exclusion. In colorectal cancer, FadA from F. nucleatum can bind E-cadherin and activate β-catenin signaling, promoting tumor-cell proliferation and an immune-excluded phenotype^[97]^. In parallel, F. nucleatum can activate TLR4/MyD88-NF-κB signaling. The coexistence of these signals suggests that the same microbial stimulus may simultaneously engage oncogenic and inflammatory pathways, although direct molecular coupling between NF-κB and β-catenin in intratumoral microbiota remains insufficiently established.

(4) cGAS-STING pathway

The cGAS-STING pathway represents an additional microbial-responsive pathway that may influence the balance between antitumor immunity and immune tolerance. Microbial or damage-associated signals can engage this pathway and promote type I interferon responses, but its functional consequences may depend on the tumor context and the intensity and duration of pathway activation. Its potential interaction with NF-κB and other inflammatory pathways further supports the concept that intratumoral microbial sensing operates through interconnected rather than isolated signaling modules^[98]^.

(5) PI3K/Akt/mTOR pathway

Microbial signals may also influence the PI3K/Akt/mTOR pathway, which regulates tumor-cell survival, metabolism, and growth. In the tumor microenvironment, convergence between microbial sensing, inflammatory signaling, and PI3K/Akt/mTOR activity may contribute to cellular adaptation and survival under metabolic or therapeutic stress. However, direct evidence defining the contribution of intratumoral microbiota to coordinated regulation of these pathways remains limited^[70,99]^.

(6) Immune checkpoint regulatory pathways

The PD-1/PD-L1 pathway is a classic mechanism of immune suppression. The NF-κB, STAT3, and MAPK pathways can all upregulate PD-L1 expression, thereby enhancing T-cell exhaustion^[50,100]^. Additionally, the Fap2 protein from *F. nucleatum* can directly bind to the TIGIT receptor, bypassing traditional signaling pathways to directly inhibit the activity of NK cells and CD8^+^ T cells, representing a receptor-level immune suppression mechanism^[50,88]^.

(7) NLRP3 inflammasome pathway

Upon sensing bacterial components, the NLRP3 inflammasome promotes caspase-1 activation and the maturation and release of IL-1β^[101]^. IL-1β enhances MDSC recruitment and promotes angiogenesis^[102]^. Chronic, low-level NLRP3 activation is associated with immune suppression in various solid tumors, suggesting that tumor-associated microbiota may enhance immune evasion by maintaining inflammasome activity^[102]^.

(8) Crosstalk among microbial-responsive signaling pathways

Microbial stimuli may not activate these signaling pathways independently but can establish functional crosstalk among them within the tumor microenvironment. In particular, activation of TLR/MyD88-NF-κB signaling can induce the production of inflammatory cytokines such as IL-6, which subsequently activates the JAK/STAT3 pathway. This provides a mechanistic link between persistent microbial sensing, chronic inflammation, and STAT3-mediated immunosuppression^[103,104]^. *Streptococcus anginosus* has also been shown to induce NF-κB activation and increase IL-6 production in macrophages, supporting a link between microbial stimulation and inflammatory signaling^[55,105]^.

The Wnt/β-catenin pathway may further converge with inflammatory signaling to promote tumor progression. In colorectal cancer, FadA from *Fusobacterium nucleatum* binds E-cadherin and activates β-catenin signaling, thereby promoting tumor cell proliferation^[106]^. Meanwhile, *F. nucleatum* can activate the TLR4/MyD88-NF-κB pathway and promote inflammatory and tumor-promoting responses^[89,107]^. These findings suggest that microbial stimuli can simultaneously engage oncogenic and inflammatory pathways, potentially producing complementary effects on tumor proliferation, inflammation, and immune regulation. However, direct evidence for coordinated regulation among NF-κB, STAT3, and Wnt/β-catenin specifically in response to intratumoral microbiota remains limited, and much of the available evidence is derived from specific tumor types and experimental models. Therefore, these pathways are best considered interconnected and potentially cooperative signaling networks rather than universally synergistic or antagonistic pathways.

## THE IMPACT OF INTRATUMORAL MICROBIOTA ON CANCER THERAPY

Intratumoral microbiota can influence responses to chemotherapy, immunotherapy, radiation therapy, and targeted therapy through mechanisms involving drug metabolism, immune regulation, oxygen availability, and host signaling. The following sections summarize the evidence for each treatment modality and highlight the current limitations of mechanistic and translational evidence.

### Regulation of chemotherapy

A growing body of research indicates that the intratumoral microbiota can significantly influence the efficacy of chemotherapy through multi-level mechanisms, including direct drug metabolism, regulation of tumor cell stress and death pathways, and remodeling of the microenvironment. This regulation can occur both before drugs enter tumor cells and at the level of intracellular signaling networks and the microenvironment, thereby contributing to the development of drug resistance. Recent preclinical evidence further suggests that modulation of the intratumoral microbiome can enhance therapeutic efficacy and microbiota-related antitumor immune responses in colorectal cancer models^[108]^.

In pancreatic ductal adenocarcinoma, a study has for the first time systematically demonstrated that γ-proteobacteria enriched within tumor tissue can express bacteriogenic cytidine deaminase (CDD), metabolizing gemcitabine into inactive metabolites^[8]^. Both in vitro cultures and mouse models demonstrated that tumors harboring these bacteria exhibit significant resistance to gemcitabine, whereas drug sensitivity is restored after the bacterial population is eradicated with antibiotics^[8]^. This finding clearly establishes that intratumoral bacteria can directly “inactivate” chemotherapeutic agents through enzymatic reactions, thereby constituting a metabolic barrier to drug resistance.

In colorectal cancer, Fusobacterium nucleatum has been shown to be associated with resistance to 5-fluorouracil and oxaliplatin. Studies have found that this bacterium can activate the TLR4/MyD88 signaling axis, further inducing the expression of autophagy-related genes (such as members of the ATG family) and enhancing autophagy flux^[65,109]^. Enhanced autophagy may allow tumor cells to maintain energy homeostasis and clear damaged organelles under chemotherapy-induced stress, thereby reducing apoptosis^[109]^. Upon removal of this bacterium, autophagy levels decrease and chemotherapy sensitivity increases, suggesting that microorganisms may contribute to chemotherapy resistance by regulating autophagy-related survival pathways. However, these findings primarily support a role for intratumoral microorganisms in modulating responses to cytotoxic chemotherapy, and whether the same mechanism contributes to resistance to targeted therapies such as cetuximab remains insufficiently established.

In addition to directly influencing drug metabolism and autophagy pathways, intracellular bacteria may also weaken the efficacy of chemotherapy by enhancing the stress adaptation capacity of tumor cells. In breast cancer and melanoma models, *in situ* studies have observed that certain bacteria can reside intracellularly within tumor cells^[9,58]^. These intracellular bacteria are believed to enhance cellular tolerance to oxidative stress and DNA damage, potentially through the upregulation of antioxidant response pathways (such as Nrf2) and DNA repair-related signaling, thereby reducing the cytotoxic effects of chemotherapeutic agents - such as anthracyclines - that rely on oxidative damage mechanisms to exert their effects^[24,58]^.

On the other hand, the tumor microbiome can also indirectly regulate the response to chemotherapy by altering the microenvironment. Persistent microbial stimulation can sustain a chronic inflammatory and immunosuppressive environment, activating pro-survival signaling pathways such as NF-κB and STAT3, increasing the expression of Bcl-2 family proteins, and raising the apoptosis threshold^[110]^. Under these conditions, tumor cells are more likely to survive and undergo selective expansion under chemotherapy stress. Furthermore, inflammation-driven infiltration of tumor-associated fibroblasts and immunosuppressive cells may further enhance drug resistance phenotypes through paracrine signaling^[110]^. Together, these findings indicate that intratumoral microbiota can influence chemotherapy response through both direct drug metabolism and indirect modulation of tumor-cell survival and the microenvironment.

### Impact on tumor immunotherapy

The role of intratumoral microbiota in immunotherapy is receiving increasing attention, with its effects spanning multiple levels, including the regulation of host immune signaling, immune cell infiltration and functional status, activation of inflammatory pathways, and drug responsiveness. Existing evidence indicates that this regulation is distinctly bidirectional: it may both attenuate the efficacy of immune checkpoint inhibitors (ICIs) and, under specific conditions, enhance antitumor immune responses^[111]^.

First, regarding immunosuppression, certain tumor-associated microorganisms can reduce the efficacy of ICIs by directly interfering with immune receptor signaling. In colorectal cancer, the enrichment of Fusobacterium nucleatum has been shown to be associated with an immunosuppressive phenotype. Its Fap2 protein binds to the TIGIT receptor on the surface of immune cells, inhibiting the cytotoxic activity of CD8^+^ T cells and NK cells, while simultaneously promoting the polarization of macrophages toward the M2 phenotype, thereby creating an immunosuppressive microenvironment. Under these conditions, the efficacy of anti-PD-1 therapy is diminished^[88,110]^. Furthermore, in breast cancer models, intratumoral bacteria have been reported to modulate inflammatory and immune-related signaling pathways, including NF-κB, which may contribute to an immunosuppressive tumor microenvironment and altered antitumor immune responses^[32,112]^.

On the other hand, evidence from gut microbiota studies indicates that certain gut microorganisms and their metabolites can enhance antitumor immune responses and improve the efficacy of ICIs. For example, specific strains of Bacteroides fragilis can modulate dendritic cell function through PSA-associated TLR2/1 and Dectin-1 signaling, thereby promoting IL-12 production and Th1-type immune responses^[113]^. In addition, its capsular polysaccharide A (PSA) has been reported to contribute to the induction of Th1-mediated immune responses through TLR2-dependent signaling^[113]^. At the metabolic level, butyrate, a short-chain fatty acid primarily produced by the gut microbiota, can enhance the metabolic fitness and memory differentiation of CD8^+^ T cells by inhibiting histone deacetylases (HDACs) and modulating the mechanistic target of rapamycin (mTOR) signaling pathway, thereby improving responses to immunotherapy^[114]^. However, these findings are derived predominantly from studies of the gut microbiota, and direct evidence demonstrating similar immunomodulatory effects of intratumoral microbiota remains limited.

In addition to direct immune regulation, microbiota can indirectly influence the efficacy of immunotherapy by affecting antigen processing and drug metabolism. In pancreatic ductal adenocarcinoma, enriched γ-proteobacteria not only express cytidine deaminase to degrade gemcitabine but may also reduce the synergistic effect of chemotherapy combined with immunotherapy by modulating the expression of molecules involved in antigen processing and presentation^[8]^. Furthermore, microbiota-induced chronic low-grade inflammation can sustainably activate STAT3, MAPK, and NF-κB signaling pathways, upregulating PD-L1 expression and thereby forming a local immunosuppressive barrier that limits the efficacy of ICIs^[115]^.

Notably, the spatial localization of intratumoral microbes may also influence the response to immunotherapy^[116]^. Intracellular bacteria may alter the efficiency of antigen presentation by tumor cells by providing bacterial-derived peptides that can be presented on MHC-I molecules^[117]^; conversely, bacteria residing within immune cells may influence their differentiation and functional polarization, thereby reshaping the structure of the immune microenvironment at the cellular level.

Overall, intratumoral microbiota exert bidirectional regulatory effects on immunotherapy through multidimensional mechanisms, including immune signaling regulation, activation of inflammatory pathways, alterations in antigen processing, and metabolism-mediated immune adaptation. Their function depends on microbial composition, abundance, spatial distribution, and the state of coupling with host signaling networks. A systematic analysis of the interaction mechanisms between intratumoral microbiota and immune pathways holds significant theoretical and clinical implications for optimizing ICI treatment strategies, predicting treatment efficacy, and developing combination therapies based on microbiome-targeted interventions.

### Impact on physical therapies and targeted therapies

In addition to chemotherapy and immunotherapy, the tumor microbiome can significantly influence the efficacy of physical therapies such as radiation therapy and photodynamic therapy (PDT) by regulating oxygen metabolism, redox balance, cellular stress networks, and inflammatory signaling pathways. It also alters the responsiveness to targeted drugs by interfering with key molecular pathways. This regulation involves both metabolic changes at the tumor microenvironment level and the reprogramming of intracellular signaling networks.

During radiotherapy, ionizing radiation relies on oxygen to generate reactive oxygen species (ROS), which in turn induce double-strand breaks and irreversible DNA damage, resulting in the oxygen enhancement effect^[118]^. Studies have confirmed that bacteria can persist stably within tumor cells. Functional experiments suggest that these intracellular bacteria enhance the host cells’ tolerance to stress and DNA damage, potentially by activating antioxidant response pathways and DNA repair-related molecules, thereby reducing radiation-induced cell death^[9]^. Furthermore, anaerobic or facultative anaerobic bacteria enriched within tumors may contribute to a hypoxic microenvironment through local oxygen consumption, potentially reducing the efficiency of oxygen-dependent ROS generation during radiotherapy^[119]^. In colorectal and pancreatic cancer tissues, anaerobic bacterial load has been reported to be positively associated with the degree of hypoxia and radiotherapy resistance, suggesting a potential role of these microorganisms in modulating the tumor response to radiotherapy^[119]^.

Similar mechanisms also influence photodynamic therapy (PDT). PDT similarly relies on oxygen-dependent photosensitizer excitation reactions to generate ROS^[120]^. Microbial metabolic activity can alter the local redox state and pH environment, and by continuously activating pro-survival pathways such as NF-κB, it enhances tumor cell tolerance to oxidative damage, thereby reducing the cytotoxic effects induced by PDT^[121]^. This microbiome-driven remodeling of the oxygen environment provides a metabolic basis for tolerance to physical therapies.

In the context of molecularly targeted therapy, intratumoral microbiota can influence drug targets and their downstream networks by modulating host signaling pathways, thereby establishing bypass activation or survival signaling maintenance mechanisms. For example, in colorectal cancer, *Fusobacterium nucleatum* has been reported to enhance autophagy flux through the TLR4/MyD88 signaling axis, which may contribute to reduced sensitivity to the anti-EGFR monoclonal antibody cetuximab. This effect is proposed to involve enhanced tumor cell survival under EGFR blockade; however, direct evidence linking *F. nucleatum*-induced autophagy to cetuximab resistance remains limited and requires further validation.

In hepatocellular carcinoma models, bacterial lipopolysaccharide (LPS) activates TLR4 signaling and enhances NF-κB and STAT3 pathway activity, which has been shown to be associated with sorafenib resistance^[36]^. Persistent activation of NF-κB and STAT3 may upregulate anti-apoptotic Bcl-2 family proteins and pro-angiogenic factors, potentially attenuating the therapeutic efficacy of inhibitors targeting the MAPK or VEGF pathways^[36]^. These findings suggest that microbial-induced innate immune receptor signaling may contribute to the maintenance of alternative survival pathways during molecularly targeted therapy.

Together, these findings suggest that microbial regulation of oxygen availability, redox balance, and host signaling may contribute to variable responses to physical and targeted therapies, although causal evidence remains limited.

## TECHNICAL CHALLENGES AND RESEARCH PROSPECTS IN INTRATUMORAL MICROBIOME RESEARCH

The primary technical challenge in tumor microbiome research stems from its “ultra-low biomass” nature and the resulting high sensitivity to contamination^[56]^. In most solid tumor samples, bacterial DNA copy numbers are far lower than the host genomic background, often approaching the lower limit of detection for sequencing, making reagent contamination, environmental exposure, or batch effects likely sources of significant bias^[56]^. Consequently, “background microbial profiles” may arise from sample collection, the selection of DNA extraction reagents, the construction of amplification systems, and the sequencing platform itself^[56]^. Distinguishing true biological signals from technical contamination under conditions of low signal-to-noise ratio has become the most challenging problem in this field^[56]^. For low-biomass tumor samples, specific contamination-control strategies should therefore be incorporated into the experimental workflow. Essential negative controls should include extraction blanks and reagent controls, with library preparation controls and environmental controls included when appropriate. Computational contaminant-identification tools, such as the R package decontam, can be used together with these controls to identify potential contaminants based on their prevalence or abundance patterns across samples and negative controls. Sequencing-based approaches primarily provide relative microbial abundance, whereas droplet digital polymerase chain reaction (ddPCR) may be considered for absolute quantification of predefined low-abundance microbial targets. However, ddPCR should not be used as a standalone method and should be combined with appropriate negative controls and orthogonal validation, such as quantitative polymerase chain reaction (qPCR), fluorescence *in situ* hybridization, or culture-based approaches when feasible. Batch tracking and standardized sample-processing procedures should also be implemented to enhance the reliability and reproducibility of results.

The second key challenge lies in obtaining localization and visualization evidence. Relying solely on 16S rRNA sequencing or metagenomic data can only indicate a “presence signal,” but it is difficult to prove that microorganisms are actually located within tumor tissue, let alone distinguish whether they are situated in the extracellular matrix, intracellularly, or within blood vessels. A study used *in situ* hybridization and electron microscopy to systematically confirm for the first time that bacteria can exist inside tumor cells and immune cells^[9]^. Subsequently, a study further validated intracellular localization using spatially resolved techniques^[24]^. However, methods such as RNAscope, 16S rRNA fluorescence *in situ* hybridization (FISH), electron microscopy, and spatial transcriptomics impose extremely high demands on sample fixation conditions, probe specificity, and signal interpretation criteria, and are difficult to implement at high throughput in large-scale clinical cohorts^[56]^.

Third, distinguishing between viable and non-viable bacteria also poses a significant challenge. Detection at the DNA level cannot determine whether microorganisms possess metabolic activity, and bacteria within tumors often exist in a state of low proliferation or dormancy^[122]^. The introduction of metatranscriptomics and metabolomics technologies has made it possible to assess microbial activity; however, poor RNA stability, time-sensitive sample processing, and extremely low culture success rates all limit functional validation in clinical samples^[56]^. Furthermore, the integration of culture-dependent and culture-independent techniques remains to be standardized.

Spatial heterogeneity, combined with the molecular heterogeneity of the tumor itself, further increases the complexity of interpretation. Microbial distributions may vary significantly across different regions (necrotic zones, hypoxic zones, invasion fronts, and perivascular zones), yet conventional tissue homogenate sequencing masks this spatial information^[56]^. Spatial omics technologies, such as spatial transcriptomics and spatial metagenomics, are being used to elucidate the local interaction networks among microbes, tumors, and immune cells; however, they are costly, have limited throughput, and involve complex data integration^[56]^. At the bioinformatics level, the overwhelming dominance of host sequences and the inadequacy of reference databases also limit accurate annotation; low-abundance microbial sequences are prone to being misfiltered or misclassified. Therefore, analytical algorithms optimized for low-biomass samples and the development of more comprehensive reference databases are key directions for future progress.

Despite these methodological challenges, the field of intratumoral microbiology holds considerable promise, but the strength of current conclusions remains uneven because many reported associations are based on low-biomass sequencing datasets without sufficient spatial or functional validation. With advancements in spatial multi-omics and single-cell technologies, interactions among microbes, tumor cells, and immune cells may increasingly be resolved at cellular resolution. However, technological advances alone will not establish causality; mechanistic validation using germ-free or gnotobiotic animal models, organoid co-culture systems, controlled microbial colonization models, and complementary molecular approaches remains essential. The standardization of sampling procedures, implementation of decontamination protocols, and integration of spatial, quantitative, and functional validation will therefore be critical for distinguishing reproducible biological signals from technical or correlative observations and for moving the field from descriptive profiling toward mechanistically supported conclusions.

## CLINICAL APPLICATIONS OF INTRATUMORAL MICROBIOME RESEARCH

As mechanistic research continues to deepen, intratumoral microbiome research is beginning to reveal potential opportunities for clinical translation, particularly in diagnostic classification, prognostic assessment, treatment-response prediction, and microbiome-based therapeutic strategies. Its value is primarily reflected in diagnostic classification, prognostic assessment, prediction of treatment response, and therapeutic intervention strategies. Despite its considerable translational potential, direct clinical evidence specifically supporting intratumoral microbiome-based diagnosis, prognostic stratification, or therapeutic intervention remains limited. Most current evidence is derived from observational studies, retrospective analyses, and preclinical models rather than prospective clinical trials. Therefore, the clinical applications discussed below should be considered emerging possibilities rather than established clinical practices.

First, at the diagnostic and classification level, relatively stable and distinguishable intratumoral microbial profiles exist across different cancer types and molecular subtypes. For example, the enrichment of *Fusobacterium nucleatum* in colorectal cancer and the high proportion of γ-proteobacteria in pancreatic ductal adenocarcinoma both demonstrate a certain degree of cancer-specificity^[123]^. Integrating microbiome data with histopathological and molecular subtyping data holds promise for improving the accuracy of subtyping and optimizing risk stratification models. However, these applications currently remain investigational and require validation in larger, prospective, multicenter cohorts.

Second, regarding prognostic assessment, the abundance levels of specific microbial communities are statistically correlated with tumor progression rates, metastasis risk, and overall survival. For instance, a study demonstrated that distinct intratumoral microbial profiles in pancreatic cancer patients are significantly associated with long-term survival, suggesting that microbiome characteristics can be incorporated into prognostic prediction systems^[10]^.

Regarding treatment response prediction, the intratumoral microbiota has emerged as a potential predictor by modulating responsiveness to chemotherapy, radiotherapy, targeted therapy, and immunotherapy^[124]^. Assessing the state of the tumor microbiome prior to treatment may predict a patient’s likelihood of responding to specific therapies, thereby aiding personalized treatment decisions^[125]^. However, evidence supporting its use for prospective treatment selection remains insufficient. Large, well-controlled clinical cohorts are needed to determine whether intratumoral microbial profiles can reproducibly predict responses to chemotherapy, radiotherapy, targeted therapy, or immunotherapy. In addition, modulation of the microbiota through antibiotics, probiotics, bacteriophages, or other microbiome-based interventions has shown potential for improving therapeutic responses. However, most of the current evidence is derived from studies of the gut microbiota, and whether these strategies can be directly applied to target the intratumoral microbiota requires further investigation^[125]^.

Moreover, certain bacteria possess tumor-targeting capabilities and hypoxic adaptation, allowing them to selectively accumulate in tumor tissues. This characteristic has been utilized to develop engineered bacterial delivery systems. For example, attenuated strains of Salmonella enterica and Clostridium novyi have been employed to deliver antitumor genes or immunostimulatory factors, enabling highly efficient local expression and selective killing, and providing a new technical pathway for biological vector therapy^[126]^. These approaches primarily represent engineered microbial therapeutics rather than direct manipulation of endogenous intratumoral microbiota, and their clinical applicability requires further validation.

In the field of minimally invasive detection, tumor-derived microbial DNA or metabolites can enter the circulatory system, offering potential as supplementary markers for liquid biopsy^[127]^. Microbial signals in blood or other body fluids may reflect changes in the tumor microenvironment, providing new tools for non-invasive monitoring of tumor progression and treatment response^[127]^.

## CONCLUSION AND OUTLOOK

Intratumoral microbiota represent a low-biomass and spatially heterogeneous component of the tumor ecosystem. Current evidence indicates that their biological effects are highly context-dependent and shaped by microbial composition, spatial localization, metabolic conditions, and host immunity. Rather than functioning as uniformly tumor-promoting or tumor-suppressive entities, intratumoral microorganisms may influence tumor behavior and treatment response through interconnected host-microbe interactions. This context-dependent perspective highlights the importance of integrating microbial characteristics with the spatial, metabolic, and immune features of the tumor microenvironment when interpreting their biological effects.

Important challenges remain, particularly in contamination control, spatial localization, functional validation, and causal interpretation. Future studies integrating standardized sampling, rigorous negative controls, spatially resolved approaches, and complementary quantitative and functional validation will be essential for distinguishing genuine microbial signals from technical artifacts and for establishing clinically relevant mechanisms. Ultimately, determining the reproducibility, causality, and clinical relevance of these microbial effects will be critical for assessing whether intratumoral microbiota can be reliably exploited as biomarkers or therapeutic targets.

## DECLARATIONS

### AI and AI-assisted tools statement

During the preparation of this manuscript, the AI tool ChatGPT (version GPT-5.5, released 2025-08-07) was used solely for language editing. The tool did not influence the study design, data collection, analysis, interpretation, or the scientific content of the work. All authors take full responsibility for the accuracy, integrity, and final content of the manuscript.

### Copyright

© The Author(s) 2026.
